# Targeting the gut-lung axis in COPD: from microbial metabolites to fecal microbiota transplantation

**DOI:** 10.3389/fmicb.2026.1798150

**Published:** 2026-04-01

**Authors:** Zaixin Yu, Weishi Qian, Yanbiao Chu

**Affiliations:** 1 Jinan Central Hospital, Shandong University, Jinan, China; 2 School of Clinical Medicine, Shandong Second Medical University, Weifang, China

**Keywords:** COPD, FMT, gut microbiota, gut-lung axis, mechanisms, microbial metabolites

## Abstract

Chronic obstructive pulmonary disease (COPD) is a complex, multidimensional syndrome manifested by persistent airway inflammation, oxidative stress, and progressive airflow limitation, with pathology extending far beyond the lung. The gut-lung axis has emerged as a pivotal paradigm for understanding this systemic nature, underscoring the regulatory potency of gut microbiota-derived metabolites in inter-organ immune and metabolic crosstalk. Accumulating evidence suggests that COPD is intricately linked to gut microbiota dysbiosis and widespread disturbances in bioactive metabolites, particularly short-chain fatty acids (SCFAs), tryptophan-related amino acids (AAs), and bile acids (BAs). These metabolic aberrations exacerbate pulmonary inflammation by dysregulating immune homeostasis, compromising intestinal barrier integrity, and skewing redox balance. Fecal microbiota transplantation (FMT), as a strategy capable of comprehensively reconstituting gut microbial and metabolic homeostasis, has demonstrated potential in preclinical and translational settings to attenuate pulmonary injury via the gut-lung axis. This review centers on gut microbiota-associated metabolites, systematically summarizing their roles in COPD pathogenesis and critically evaluating the emerging evidence and mechanistic basis by which FMT recalibrates COPD progression through metabolic pathways, thereby providing a robust theoretical framework for developing precision gut microbiota–targeted systemic therapeutic strategies.

## 1 Introduction

Chronic obstructive pulmonary disease (COPD) is a life-threatening chronic inflammatory airway disorder predominantly triggered by prolonged exposure to noxious stimuli, particularly cigarette smoke, and is characterized by persistent respiratory symptoms and irreversible, progressive airflow limitation (Rabe and Watz, 2017; Labaki and Rosenberg, 2020). As a major global public health challenge, COPD is expected to impose an increasing burden in the context of population ageing and persistent environmental pollution. The global prevalence is projected to reach approximately 600 million by 2050, placing substantial economic pressure on healthcare systems and remaining a leading contributor to disability-adjusted life years (DALYs) and mortality worldwide (Boers et al., 2023; Chen et al., 2023). Beyond localized pulmonary pathology, COPD is now recognized as a complex systemic syndrome, frequently accompanied by cardiovascular diseases, metabolic abnormalities, and skeletal muscle dysfunction (Barnes, 2010). These systemic features suggest that the initiation and progression of COPD are not confined to localized pulmonary pathology but instead involve dysregulation of complex, multi-level, and inter-organ regulatory networks. This highlights the urgency of unraveling their systemic mechanisms to develop effective therapeutic interventions.

In recent years, the paradigm of the “gut-lung axis” concept has revolutionized our understanding of COPD’s systemic pathological features, offering a pivotal integrative framework that bridges local pulmonary pathology with systemic regulatory networks. The gut and the lung are interconnected through a tightly coordinated bidirectional communication network involving immune regulation, metabolic homeostasis, barrier integrity, and neuroendocrine signaling pathways (Enaud et al., 2020; Liu et al., 2026; Dang and Marsland, 2019). As a central regulatory hub within the gut-lung axis, the maintenance of gut microbial homeostasis, or its disruption, exerts a decisive influence on pulmonary immune responses, inflammatory cascades, and tissue repair processes via these interconnected pathways, thereby dictating lung health trajectories and the progression of COPD (Wei et al., 2022; Xu et al., 2024; Song et al., 2024). Multiple basic research and clinical observational investigations have reported varying degrees of gut microbiota compositional alterations in patients with COPD (Viglino et al., 2026; Bowerman et al., 2020; Li et al., 2021). This dysbiotic state is not merely confined to altered abundance of individual microbial taxa but typically involves aberrant fluctuations across multiple taxonomic levels, accompanied by a marked decline in overall microbial diversity, including both species richness and evenness, which further impairs the regulatory capacity of the gut-lung axis. Notably, alterations in microbial community structure are frequently coupled with profound shifts in metabolic profiles, characterized by dysregulated metabolite biosynthesis, secretion, and downstream metabolic pathway activity, changes that propagate through the gut-lung axis to exacerbate pulmonary pathology (van Iersel et al., 2025; Budden et al., 2017; Dowling et al., 2025).

Against this backdrop, strategies aimed at modulating the gut micro-ecosystem to intervene in systemic inflammation and disease progression in COPD have emerged as a research focus. Fecal microbiota transplantation (FMT), as an intervention that globally reshapes the structure and function of the gut microbial community, has demonstrated promising therapeutic potential in a range of microbiota-associated diseases in recent years (Minkoff et al., 2023; Jayachandran and Qu, 2023; Ma et al., 2024). However, in the context of COPD, research on FMT remains at an exploratory stage. Available evidence suggests that FMT may indirectly ameliorate pulmonary inflammation and function by re-establishing gut microbial homeostasis and recalibrating dysregulated metabolic pathways (Zhang et al., 2025; Budden et al., 2024). Nevertheless, the key mediating metabolites and their underlying mechanisms have not yet been systematically integrated, nor are they supported by sufficient robust clinical and preclinical evidence. Previous reviews in this field have mostly focused on differences in gut microbiota composition or macroscopic crosstalk along the gut-lung axis (Karakasidis et al., 2023; Lim et al., 2023; Song et al., 2023), while the integration of evidence specifically focusing on gut microbial metabolites as critical mediating factors remains inadequate.

Accordingly, this review aims to systematically synthesize the latest literature in re-cent years, with gut microbiota-derived metabolites as the unifying framework. We dissect their molecular roles in regulating pulmonary inflammation, immune responses, and oxidative stress in COPD, critically evaluate the current advances and potential mechanistic pathways of FMT-based interventions, and ultimately provide a mechanistically grounded theoretical basis and conceptual framework for the development of gut microbiota-targeted therapeutic strategies for COPD.

## 2 Systemic features of COPD and the basis of the gut-lung axis

The core pathophysiological features of COPD encompass persistent airway inflammation, excessive oxidative stress, and progressive structural damage to lung tissue. However, a growing body of evidence indicates that COPD is not confined to a localized pulmonary disorder; rather, its initiation and progression are tightly linked to systemic inflammatory responses and perturbations in metabolic homeostasis (Brightling and Greening, 2019; Wang et al., 2023). Prolonged exposure to noxious stimuli, including cigarette smoke, biomass fuel combustion products, and ambient air pollutants, is considered the key initiating driver of COPD pathological progression. These insults trigger sustained inflammatory responses in the airways and lung parenchyma, ultimately leading to hallmark pathological alterations such as small airway remodeling, alveolar destruction, and progressive airflow limitation (Agustí et al., 2023). During this process, inflammatory cells, predominantly neutrophils and macrophages, infiltrate the lung tissue, accompanied by sustained secretion of pro-inflammatory cytokines, including tumor necrosis factor-*α* (TNF-α) and interleukin-6 (IL-6). Inflammatory responses and oxidative stress form a mutually reinforcing vicious cycle, which exacerbates lung tissue injury and accelerates disease progression (Shi et al., 2024). Meanwhile, inflammatory mediators and oxidative products generated in the lungs can enter the systemic circulation, triggering a state of chronic, low-grade systemic inflammation that facilitates the development and progression of multiple extrapulmonary comorbidities, including cardiovascular diseases, immune dysregulation, skeletal muscle wasting, and metabolic abnormalities (Patraș et al., 2025; Hurst and Sin, 2018; Parpaleix et al., 2017).

Against this background, the gut-lung axis has generated increasing attention as a critical inter-organ regulatory pathway linking the gut microecosystem to pulmonary immune homeostasis. Current evidence indicates that the function of the gut-lung axis is dependent on the synergistic interplay of multiple interconnected signaling pathways. When intestinal barrier integrity is impaired, gut microorganisms and their structural components can translocate into the systemic circulation and then migrate to lung tissue, where they activate alveolar macrophages and other innate immune cells, thereby initiating or amplifying local inflammatory cascades in the lung (Moon et al., 2025; Nascimento et al., 2023). In parallel, the gut microbiota modulates systemic immune response profiles by regulating the differentiation and functional phenotypes of immune cells within gut-associated lymphoid tissue (GALT). Once “programmed” by the gut microbiota, these immune cells can migrate to the lung and exert sustained regulatory effects on the magnitude and quality of local inflammatory responses (Xu et al., 2024). In addition, gut-derived microbial signaling molecules and metabolites can be transported via the bloodstream to the lung, where they directly or indirectly modulate the homeostasis of the pulmonary immune microenvironment. For example, gut-derived lipopolysaccharide (LPS) and short-chain fatty acids (SCFAs) can act on pulmonary cells to reprogram the immunometabolic state of alveolar macrophages, thereby contributing to the establishment of basal pulmonary immune tone (Liu et al., 2021). Tryptophan-derived metabolites, such as indole-3-acetic acid, can regulate the extent of tissue injury and the dynamics of oxidative stress responses in the lung during infection through aryl hydrocarbon receptor (AhR)-dependent signaling pathways (Kullberg et al., 2025). Notably, the potential role of bile acids (BAs) in gut-lung axis regulation has garnered growing interest in recent years. However, existing studies have largely focused on bile acid receptor-mediated signaling and acute inflammatory regulation. In the context of COPD, BA metabolic dysregulation and its molecular mechanisms remain insufficiently supported by direct and systematic experimental and clinical evidence, warranting further in-depth investigation (Dowling et al., 2025). Overall, the gut-lung axis provides a crucial conceptual framework for understanding the systemic pathological features of COPD and lays the foundation for subsequent mechanistic analyses at the level of microbial metabolites.

## 3 Gut metabolic dysregulation and mechanisms in COPD

**Figure 1:**
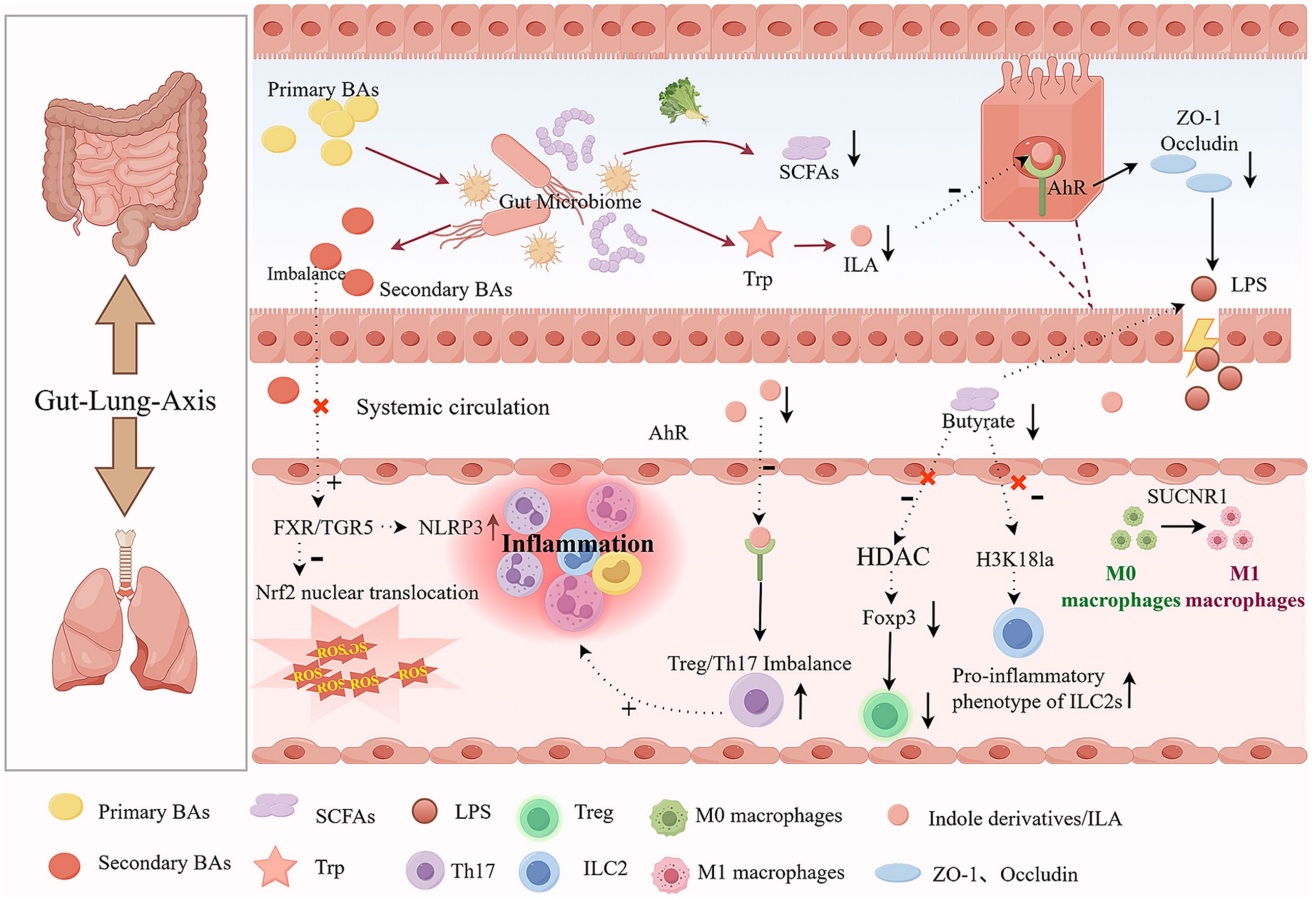
Gut-lung axis dysregulation in COPD: microbial metabolite imbalance drives pulmonary inflammation and immune dyshomeostasis (by Figdraw.) (Illustrates the pathological cascade by which gut microbial dysbiosis and metabolite imbalance disrupt gut-lung axis homeostasis, exacerbating COPD pathophysiology).

Notably, gut microbiota functional dysbiosis associated with COPD is characterized by systemic perturbations in multiple classes of gut-derived metabolites. These metabolic abnormalities are not isolated events but are propagated via gut-lung axis-mediated interorgan signaling, thereby contributing to immune dysregulation, amplification of inflammatory responses, barrier dysfunction, and enhanced oxidative stress. As shown in Table 1 and Figure 1, which illustrates the pathological cascade by which gut microbial dysbiosis and metabolite imbalance disrupt gut-lung axis homeostasis, exacerbating COPD pathophysiology. The following sections will focus on several representative metabolic pathways, such as SCFAs, AAs, and BAs, systematically describing their dysregulation and potential pathogenic roles in COPD.

**Table 1:** Impact mechanisms of gut metabolites or their dysregulation on disease.

Category	Specific metabolites	Role or effect in disease	References
SCFAs	Butyrate	Enhances H3K27 acetylation at the Foxp3 promoter/CNS1 by inhibiting HDAC, promoting Treg differentiation	Arpaia et al. (2013)
		Inhibits ILC2 expansion and H3K18la lactylation, reducing their pro-inflammatory phenotype	Jiang et al. (2023), Jiang et al. (2026)
		Intestinal epithelial energy substrate, maintaining barrier homeostasis	Zhao et al. (2018), Kelly et al. (2015), Lai et al. (2022)
	Acetate	Regulates alveolar macrophage immunometabolism via FFAR2/FFAR3	Liu et al. (2021)
		Reprograms macrophage glycolysis, activating the HIF-1α/NLRP3 axis and upregulating IL-1β/NO	Machado et al. (2022)
	Propionate	Enhances intestinal tight junctions to maintain barrier integrity, suppresses pro-inflammatory signals, and modulates oxidative stress	Tong et al. (2016)
	Total SCFAs ↓	Weakens antioxidant enzyme activity and free radical scavenging, amplifying oxidative stress-related lung injury	Pang et al. (2025), Dong et al. (2025), Ferrer et al. (2024)
AAs	Trp and its indole derivatives (e.g., ILA, FICZ) ↓	Attenuates AhR signaling, downregulates expression of ZO-1 and occludin, and disrupts intestinal tight junctions	Gao et al. (2018), Wu et al. (2025)
		Impairs mucosal barrier protection via the AhR-IL-22 axis, increasing intestinal permeability and facilitating translocation of inflammatory mediators along the gut-lung axis	Lv et al. (2025)
		Weakens endogenous antioxidant defenses and free radical scavenging capacity, thereby amplifying oxidative stress-related lung tissue injury	Roager and Licht, 2018
	Kyn↑	Disrupts the Treg/Th17 balance, with increased Th17 cells and IL-17, thereby amplifying inflammation	Wang et al. (2023), Roager and Licht (2018), Prause et al. (2004), Christenson et al. (2019), Zinellu et al. (2018)
	Trp-Mel metabolites (e.g., L-Trp-NH₂, 5-HTP) ↓	Reduces 6-OH-Mel and taurine levels, promotes M1 macrophage polarization, and exacerbates oxidative stress–induced injury	Liu et al. (2024)
	Other AAs and derivatives (e.g., PAG, TrpAm, PEA) dysregulated	Remodels systemic metabolic-inflammatory networks, worsening disease and comorbidities via impairing insulin sensitivity and oxidative stress	Cao et al. (2024), Zhai et al. (2023), Wang et al. (2024)
BAs	Total BAs dysregulated	Relieves macrophage NLRP3 inhibition and alters L cell GLP-1, amplifying metabolic inflammation	Zeng et al. (2024), Guo et al. (2016)
		Weakens FXR-mediated NF-κB inhibition, increasing pro-inflammatory cytokine secretion	Gadaleta et al. (2011)
		Increases gut permeability, promoting translocation of bacterial products and inflammatory mediators to the lungs	Du et al. (2023), Zang et al. (2025), Fu et al. (2022)
	Hydrophilic BAs (e.g., UDCA) ↓	Weakens ROS clearance, Nrf2-dependent antioxidant defenses, and mitochondrial stability, amplifying oxidative stress-related inflammation and cell damage	Huang et al. (2022), Xiang et al. (2023)
MIs	Succinate ↑	Drives alveolar macrophages toward M1 polarization via SUCNR1, amplifying pulmonary inflammation	Wang et al. (2023)
NCMs	TMAO	Associated with long-term mortality in AECOPD patients, primarily reflecting comorbidities and systemic metabolic status	Ottiger et al. (2018)
NISM	GABA	Sensed by macrophages via GABA_A receptors, modulating mitochondrial metabolism and Tet2-dependent epigenetic programs to maintain antiviral defense and immune homeostasis	Gao et al. (2024)
	AI-2 dysregulated	Reflects microbial functional status and amplifies inflammation via the gut-lung axis under infection or stress	Zeng et al. (2023)
LDMs	12,13-diHOME↑	Disrupts pulmonary Treg-mediated immune tolerance, increasing airway inflammation susceptibility	Levan et al. (2019)
	DAG、S1P dysregulated	Associated with COPD symptom burden, especially in frequent exacerbators, potentially sustaining pulmonary inflammation and immune dysregulation	Ding et al. (2024)
FAs and MIs	MCFAs ↑	Reflects systemic energy metabolism remodeling	Casadevall et al. (2025)
	ACs↑		
	LCFAs, VLCFAs↓		

### 3.1 Short-chain fatty acids

Among these representative gut-derived metabolites, SCFAs, predominantly acetate, propionate, and butyrate, are key bioactive metabolites produced by the gut microbiota via the fermentation of dietary fiber (Cummings et al., 1987; Wong et al., 2006). Beyond serving as a primary energy source for intestinal epithelial cells, SCFAs also function as signaling molecules via activation of G protein-coupled receptors (GPRs), including GPR41, GPR43, and GPR109A, thereby exerting broad regulatory effects on host immune responses, inflammatory processes, and energy metabolism (Brown et al., 2003; Bolognini et al., 2016). Notably, accumulating clinical and preclinical evidence underscores that overall SCFA levels are reduced in patients with COPD. This reduction is consistently observed across multiple biological matrices and is closely correlated with disease progression. Fecal-based studies have revealed that as COPD advances from early stages to GOLD stages III-IV, the levels of total SCFAs and key components (e.g., acetate and isobutyrate) decrease significantly, suggesting that gut microbiota-derived metabolic capacity is compromised as disease severity escalates (Li et al., 2021). Comparative analyses further revealed that fecal SCFA levels are markedly lower in patients with emphysema than in asymptomatic smokers (Lee et al., 2023), supporting the notion that SCFA depletion is associated not only with COPD severity but also with specific disease phenotypes. Beyond luminal metabolic alterations in the gut, reduced circulating SCFA levels are also tightly linked to COPD and its major risk factors. Otake et al. (2025) demonstrated decreased plasma total SCFA levels, including propionate, in both smokers and patients with COPD compared with non-smokers. Notably, plasma propionate concentrations were positively associated with forced expiratory volume in 1 s (FEV₁) % predicted. Complementary animal experiments further showed that chronic cigarette smoke exposure induces gut microbiota dysbiosis accompanied by reduced fecal SCFA levels, suggesting that smoking may contribute to systemic SCFA disruption by impairing gut microbial ecology and metabolic function, thereby participating in COPD development and progression. Notably, the association between SCFAs and lung function appears to exhibit heterogeneity across different COPD phenotypes. Lee et al. (2023) further reported that, despite an overall reduction in fecal SCFA levels among patients with emphysema, residual acetate levels were strongly positively correlated with FEV₁. These findings imply that in advanced disease stages or specific phenotypes, individual SCFA components (e.g., acetate) may exert distinct biological effects compared with total SCFA levels, and may not only partially reflect lung function status but also exert compensatory protective effects. Collectively, current evidence supports a systemic decline in SCFA levels in COPD. Fecal SCFAs primarily mirror gut microbial metabolic output, whereas plasma SCFAs reflect systemic exposure to these metabolites. Together, these metrics underscore the potential role of gut-lung axis-associated metabolic imbalance in the pathophysiology of COPD.

The reduction of SCFA levels exerts multifaceted effects on COPD-associated immune dysregulation. In direct pulmonary immune regulation, butyrate inhibits histone deacetylase (HDAC) activity, enhancing histone H3 lysine 27 (H3K27) acetylation at the forkhead box P3 (Foxp3+) promoter and conserved non-coding sequence 1 (CNS1), thereby promoting peripheral differentiation of regulatory T cells (Tregs) (Arpaia et al., 2013). Besides, SCFAs, particularly butyrate, can limit the expansion and function of innate-like group 2 innate lymphoid cells (ILC2s), maintaining pulmonary immune homeostasis. A decline in SCFA levels may weaken this inhibitory effect (Jiang et al., 2023). In a cigarette smoke-induced COPD mouse model, butyrate treatment markedly reduced ILC2 cell numbers in lung tissue (Jiang et al., 2026). Furthermore, butyrate specifically suppressed histone H3 lysine 18 lactylation (H3K18la) within pulmonary ILC2s. This indicates that butyrate’s regulation of ILC2s extends beyond altering cell abundance, involving intrinsic epigenetic reprogramming that suppresses pro-inflammatory function by reducing H3K18la levels. Moreover, SCFAs such as acetate can remodel macrophage glycolysis, activate the hypoxia-inducible factor 1α (HIF-1α) axis and NOD-, LRR- and pyrin domain-containing protein 3 (NLRP3) inflammasome, thereby increasing interleukin-1β (IL-1β) and nitric oxide production and enhancing pulmonary innate immunity (Machado et al., 2022). In addition, SCFAs can modulate the metabolic state of alveolar macrophages under LPS exposure, affecting IL-1β and its receptors, free fatty acid receptor 2/3 (FFAR2/FFAR3) in lung tissue, collectively shaping pulmonary immune tone and influencing responses to subsequent infection or injury (Liu et al., 2021).

In the context of indirect gut-lung axis regulation, reduced SCFA levels impair intestinal barrier function. Studies indicated that butyrate, as a primary energy source for intestinal epithelial cells, promotes tight junction protein expression and maintains barrier integrity (Zhao et al., 2018; Kelly et al., 2015). Decreased butyrate levels may increase intestinal permeability, allowing bacterial products such as LPS to translocate via the gut-lung axis into the circulation, thereby exacerbating pulmonary inflammation (Lai et al., 2022). Additionally, propionate has also been shown to enhance tight junction protein expression, suppress pro-inflammatory cytokine release, and regulate oxidative stress, thereby improving intestinal barrier function, reducing inflammation and oxidative damage, and mitigating colitis pathology (Tong et al., 2016). These findings suggest that SCFA depletion can concurrently impair pulmonary innate immune regulation and compromise intestinal barrier integrity, disrupting gut-lung ax-is-mediated immune homeostasis and amplifying COPD-related inflammatory responses at multiple levels.

Building on the aforementioned immune dysregulation in COPD, SCFA depletion may further exacerbate disease pathology by dysregulating oxidative stress pathways. Oxidative stress is widely acknowledged as a central driver of COPD initiation and progression (Pang et al., 2025). Prior studies have demonstrated that SCFAs can alleviate oxidative damage by upregulating antioxidant enzyme activity or directly scavenging free radicals (Dong et al., 2025; Ferrer et al., 2024). Consequently, persistent depletion of intestinal SCFAs in COPD patients may compromise systemic antioxidant capacity, rendering lung tissue more vulnerable to oxidative injury. These observations highlight that SCFA levels exert a critical regulatory role in oxidative stress-associated metabolic dysregulation, thereby reinforcing their involvement in COPD pathophysiology.

### 3.2 Amino acids: focus on tryptophan

Beyond SCFAs, dysregulation of AA metabolism constitutes another hallmark of gut microbiota dysfunction in COPD patients, and this perturbation is tightly linked to disease progression (Zhang et al., 2025; Shi et al., 2024). Among AAs, tryptophan (Trp) and its metabolic pathways have become prominent research foci in the context of COPD, owing to their critical roles in preserving gut barrier integrity, regulating immune responses, and suppressing inflammatory processes (Wang et al., 2023; Agus et al., 2018). Dietary tryptophan can be metabolized by gut microbes into various bioactive molecules, such as indole derivatives (IAA, IPA, IAld) and kynurenine (Kyn) pathway products. These metabolites act as ligands for targets including the AhR, thereby modulating gut barrier function and systemic immune homeostasis (Wang et al., 2024). Approximately 20% of differentially expressed metabolites in COPD patients are closely linked to AA metabolism (Bowerman et al., 2020). Although Try metabolism was not the primary focus, these findings provide critical leads for investigating the regulatory mechanisms of specific AAs in COPD. Zhang et al. (2025) demonstrated that *Cordyceps militaris* alleviates COPD-related pathology by modulating AA metabolic profiles, restoring gut microbiota balance, and increasing SCFA levels, indirectly confirming the pivotal role of AA metabolism in disease progression. Shi et al. (2024) observed a significant reduction in plasma alanine levels in patients with acute exacerbation of chronic obstructive pulmonary disease (AECOPD), which is closely associated with dysregulated linoleic acid metabolism and alanine-specific metabolic pathways. This further highlights the involvement of AA metabolic disturbances across different stages of COPD pathophysiology.

Specifically, Trp metabolic disruption in COPD primarily impairs gut barrier integrity, disturbs systemic immune homeostasis, and exacerbates oxidative stress responses. Microbiota-derived indole metabolites can activate AhR to stabilize apical junctional complexes in intestinal epithelial cells, thereby enhancing gut barrier function and preventing abnormal increases in intestinal permeability (Gao et al., 2018). Mechanistically, indole-3-lactic acid (ILA) activates the AhR pathway, which markedly upregulates the expression of tight junction proteins Zonula occludens-1 and Occludin in Caco-2 cells, ultimately strengthening gut barrier function and reducing intestinal permeability. This protective effect was completely abrogated by the AhR antagonist CH223191 in both *in vitro* and *in vivo* experimental settings, confirming AhR as a critical mediator of gut barrier restoration (Wu et al., 2025). Moreover, gut microbiota-derived metabolites regulate mucosal immunity via the AhR-IL-22 axis, highlighting the potential role of AhR in orchestrating barrier integrity and immune homeostasis along the gut-lung axis (Lv et al., 2025). In COPD patients, gut dysbiosis may reduce the production of these protective Trp metabolites, thereby compromising in-testinal barrier integrity. This pathological alteration facilitates the translocation of bacterial products and inflammatory mediators into the blood circulation, which in turn amplifies pulmonary inflammation and oxidative stress-induced damage via gut-lung axis signaling (Chotirmall et al., 2017).

At the immune regulation level, Trp metabolites serve as key endogenous ligands for the AhR. A deficiency in these metabolites can weaken AhR signaling, thereby disrupting the balance of Treg and T helper 17 cells (Th17) pathways. This manifests as reduced differentiation and function of Tregs, particularly Foxp3⁺ and retinoic acid receptor-related orphan receptor γt (RORγt⁺) Tregs in the gut, while relieving the inhibition on Th17 cells and pro-inflammatory factors such as IL-17, ultimately leading to an imbalanced and amplified inflammatory response (Wang et al., 2023; Roager and Licht, 2018; Prause et al., 2004). Notably, AhR regulation of the Treg/Th17 axis is highly ligand- and context-dependent. Different ligands elicit varying activation intensity and duration of AhR, triggering distinct transcriptional programs and leading to differential effects on Treg/Th17 modulation. Specifically, exogenous stable ligands such as TCDD (2,3,7,8-tetrachlorodibenzo-p-dioxin) typically induce immune tolerance-related transcriptional programs across diverse experimental models, whereas high-affinity endogenous ligands like FICZ (6-formylindolo [3,2-b] carbazole) preferentially promote Th17 cell differentiation under distinct inflammatory contexts (Ehrlich et al., 2018; Ho and Steinman, 2008). In the context of COPD, Trp metabolism via the host Indoleamine 2,3-dioxygenase 1 (IDO1)-Kyn pathway and diverse microbiota-derived indole metabolites continuously supply endogenous AhR ligands. These physiologically low-toxicity ligands, upon AhR activation, tend to induce transcriptional programs that maintain mucosal homeostasis and immune tolerance, including stabilization of Treg phenotypes, enhancement of the IL-22-mediated barrier protection axis, and suppression of excessive Th17-driven inflammation, thereby functionally biasing AhR signaling toward immune protection in this context (Zelante et al., 2013; Nguyen et al., 2014; Mezrich et al., 2010). Given that the Th17/IL-17 axis contributes to airway inflammation and disease progression in COPD, and that Trp-Kyn pathway activation (e.g., increased Kyn/Trp ratio) is repeatedly observed in this population, dysregulated gut amino acid, particularly Trp metabolism, may alter the composition and signaling strength of AhR ligands, thereby disrupting Treg/Th17 homeostasis and further amplifying bidirectional interactions between systemic and pulmonary inflammation (Christenson et al., 2019; Zinellu et al., 2018).

Moreover, depletion of Trp metabolites may compromise the host’s antioxidant and anti-inflammatory defensive capacities. Accumulating evidence indicates that various indole-derived Trp metabolites exhibit intrinsic antioxidant and free radical-scavenging activities (Roager and Licht, 2018). Therefore, diminished biosynthesis of these metabolites can exacerbate oxidative stress-mediated tissue injury. Liu et al. (2024) demonstrated that enhanced biosynthesis of L-tryptophanamide (L-Trp-NH₂), 5-hydroxy-L-tryptophan (5-HTP), and 3-sulfo-L-alanine can augment the tryptophan-melatonin (Trp-Mel) pathway, elevate the concentrations of 6-hydroxymelatonin and taurine in lung tissue, and ultimately inhibit M1 macrophage polarization, thereby mitigating oxidative stress-induced damage in a murine model of COPD. Collectively, these findings confirm that perturbed Trp metabolism exerts a pivotal pathophysiological role in the onset and progression of oxidative injury in COPD. Taken together, these lines of evidence underscore that the gut microbiota-tryptophan metabolite axis holds considerable potential as a target for modulating oxidative stress and inflammatory responses in COPD.

Beyond Trp metabolites, dysregulation of other AA metabolic pathways may similarly impair the host’s capacity to modulate COPD-related pathological processes. A potential causal relationship between phenylacetylglutamine (PAG) and COPD was identified, suggesting that disrupted metabolic homeostasis of this compound may contribute to disease progression and represent a potential metabolic intervention target (Cao et al., 2024). Studies by Zhai et al. (2023) and Wang et al. (2024) also indicated that various gut microbiota-derived AA metabolites, including tryptamine (TrpAm), phenylethylamine (PEA), and branched-SCFAs, were closely associated with systemic metabolic homeostasis, inflammatory status, and oxidative stress. Imbalances in these metabolites may directly or indirectly affect insulin sensitivity, glucose regulation, and systemic inflammatory responses. Accordingly, AA metabolic dysregulation in COPD patients may not only exacerbate intrinsic pulmonary inflammation and oxidative damage but also disrupt systemic metabolic networks, potentially promoting or aggravating comorbidities such as metabolic syndrome.

### 3.3 Bile acids

BAs represent a class of steroid-derived molecules endogenously synthesized in the liver and subsequently subjected to structural modification by the gut microbiota. They exert essential roles in lipid digestion and absorption, cholesterol homeostasis, as well as the modulation of host metabolic and immune responses (Hofmann, 1999; Ridlon et al., 2006; Houten et al., 2006; Makishima et al., 1999). Via enzymatic reactions including deconjugation, dehydroxylation, and redox processes, the gut microbiota transforms hepatically derived primary BAs into secondary BAs, thereby establishing a complex and dynamic BA pool (Song et al., 2019; Funabashi et al., 2020; Marion et al., 2018). These BAs, particularly secondary BAs, function as signaling molecules by activating receptors such as the farnesoid X receptor (FXR) and Takeda G protein–coupled receptor 5 (TGR5), thereby fine-tuning inflammatory, immune, and metabolic cascades (Xiao et al., 2025; Zeng et al., 2024). In patients with COPD, gut microbiota dysbiosis is likely to induce marked perturbations in BA metabolic profiles. Prior studies have documented pervasive lipid metabolic abnormalities in COPD patients, including a marked reduction in intestinal long-chain dicarboxylic acids (Bowerman et al., 2020). Given that BAs are integral components of lipid metabolic pathways, the dysregulation of BA metabolism in COPD is biologically plausible. Furthermore, accumulating evidence indicates that BAs and their metabolites serve as central regulators of intestinal and systemic immune homeostasis, with their dysregulation being tightly correlated with the pathogenesis of inflammatory disorders (Campbell et al., 2020; He et al., 2025; Zheng et al., 2024; Zhang et al., 2025). Collectively, these findings establish a critical theoretical framework for deciphering the potential implications of BAs in COPD-associated systemic inflammation.

Dysregulation of BA metabolism can contribute to COPD-related inflammation and oxidative stress through multiple mechanisms, with its core effects manifested as aberrant inflammatory signaling, exacerbated oxidative stress, and impairment of intestinal barrier integrity. First, at the level of inflammatory signaling, BAs modulate immune responses through activation of the FXR and the bile acid receptor TGR5 (Xiao et al., 2025; Guo et al., 2016). Activation of FXR generally exerts anti-inflammatory effects, including suppression of the nuclear factor-κB (NF-κB) signaling pathway and reduction of pro-inflammatory cytokine production (Gadaleta et al., 2011). In contrast, the immunomodulatory effects of TGR5 are highly dependent on cell type and microenvironment: TGR5 activation suppresses NLRP3 inflammasome activity in macrophages (Guo et al., 2016), whereas in intestinal L cells, it indirectly regulates metabolic inflammation by promoting glucagon-like peptide-1 (GLP-1) secretion (Zeng et al., 2024). In the pathological context of COPD, gut microbial dysbiosis may alter the composition and concentrations of second BAs (e.g., deoxycholic acid, lithocholic acid), disrupting FXR- and TGR5-mediated signaling balance to amplify inflammation and exacerbate pulmonary inflammatory injury. Second, with respect to oxidative stress regulation, dysregulated BA metabolism also affects the coordinated roles of nuclear receptors in controlling oxidative stress and inflammation. A study by Huang et al. (2022) demonstrated that hydrophilic BAs, particularly ursodeoxycholic acid (UDCA) and its conjugates, can alleviate oxidative damage through multiple coordinated mechanisms, including direct scavenging of reactive oxygen species, activation of Nrf2-dependent endogenous antioxidant defenses, stabilization of mitochondrial function, and suppression of oxidative stress-associated inflammatory and apoptotic signaling. Consistently, BA-activated FXR promotes nuclear translocation of Nrf2, leading to enhanced expression of antioxidant genes (Xiang et al., 2023). In addition, BAs are essential for maintaining gut microbial homeostasis and intestinal epithelial integrity. Disruption of BA metabolism can impair epithelial tight junctions, increase intestinal permeability, and consequently facilitate the systemic translocation of bacterial products and inflammatory mediators (Du et al., 2023; Zang et al., 2025; Fu et al., 2022). Through bidirectional crosstalk along the gut-lung axis, this dysregulated process can facilitate the translocation of bacterial products and pro-inflammatory mediators to the pulmonary compartment, thereby exacerbating the inflammatory burden within the distal pulmonary parenchyma. This axis-mediated pathological cascade underscores the critical role of gut microbiota-modulated BA metabolism as a bridging link between intestinal barrier dysfunction and the perpetuation of pulmonary inflammation in COPD.

Taken together, dysregulation of BA metabolism can synergistically amplify the inflammatory and oxidative stress burdens associated with COPD by simultaneously disrupting FXR/TGR5-mediated inflammatory signaling, impairing Nrf2-dependent antioxidant defenses, and compromising intestinal barrier integrity. Within the framework of the gut-lung axis, this metabolic disturbance may represent a critical metabolic-immune hub linking gut microbiota dysbiosis to chronic pulmonary inflammation.

### 3.4 Other metabolic disturbances and multiaxial effects

Beyond the dysregulation of SCFA, AA, and BA metabolism, accumulating evidence highlights that gut microbiota dysfunction associated with COPD is also accompanied by perturbations in non-classical metabolites and signaling molecules. These include metabolic intermediates, nitrogen-containing metabolites (NCMs), neuroimmune-related small molecules (NISMs), lipid derivatives, as well as various fatty acids (FAs) and their corresponding metabolic intermediates (MIs). These bioactive molecules often exhibit marked fluctuations under conditions of stress, infection, barrier disruption, or inflammatory amplification. They may also enter the blood circulation or reprogram the metabolic pathways of immune cells, thereby contributing to pulmonary inflammatory responses and the regulation of immune homeostasis. Although systematic validation of their complete causal pathways in the context of COPD remains scarce, existing evidence provides critical clues for constructing a conceptual framework linking gut-derived metabolic signals to pulmonary effector responses.

Succinate, traditionally regarded as a tricarboxylic acid (TCA) cycle intermediate, has recently been recognized as an immunometabolic signaling molecule involved in the regulation of inflammation. Wang et al. (2023) reported that in a model of intestinal ischemia–reperfusion-induced acute lung injury, succinate accumulated markedly in lung tissue and was associated with dysregulated gut microbiota metabolism. Mechanistically, succinate can activate alveolar macrophages via its receptor succinate receptor (SUCNR1), promoting M1 polarization and thereby amplifying pulmonary inflammatory responses. This finding suggests that in the context of COPD, the spillover of gut-derived metabolic intermediates resulting from dysregulated intestinal metabolism may act as an amplifier of pulmonary inflammation. This provides important experimental evidence for understanding the metabolite-immune amplification mechanism mediated by the gut-lung axis. Meanwhile, trimethylamine N-oxide (TMAO), as a representative nitrogen-containing metabolite produced by gut microbial metabolism of dietary choline and L-carnitine, was found to be significantly elevated in a prospective study of patients with acute exacerbations of COPD, and its levels correlated positively with long-term mortality risk. However, this association appears to be largely influenced by comorbidities such as age, chronic kidney disease, and diabetes, rather than by COPD per se. TMAO primarily reflects systemic metabolic and cardiovascular risk profiles and may serve as a prognostic biomarker, but it is not suitable as an independent predictive factor. In the future, modulating TMAO levels through dietary or microbiota-targeted interventions may represent a potential strategy to improve prognosis in COPD patients (Ottiger et al., 2018).

Beyond conventional nutritional metabolites, the gut microbiota can generate signaling molecules that form inter-organ immune communication networks. *γ*-Aminobutyric acid (GABA), as a representative neuro-immune metabolite, has been shown to be produced by specific gut microbes such as *Bacteroides* species (Strandwitz et al., 2019). In a chronic stress model combined with influenza virus infection, stress-induced reductions in gut *Lactobacillus* abundance are associated with decreased GABA levels (Gao et al., 2024). Mechanistically, GABA is sensed by alveolar macrophages via GABA_A receptors and modulates mitochondrial metabolism and epigenetic programs, such as the Tet2-dependent (Ten-eleven translocation methylcytosine dioxygenase 2-dependent) pathway, to maintain cellular homeostasis, thereby enhancing pulmonary antiviral defense and mitigating inflammatory injury. In addition, bacterial quorum-sensing molecules, such as autoinducer-2 (AI-2), serve as key indicators of gut microbiota functional status. Accumulating evidence suggests that fecal AI-2 levels are correlated with inflammatory markers in patients with pneumonia and may modulate distal pulmonary immune responses by acting through the gut-lung axis (Zeng et al., 2023). In animal models, exogenous AI-2 enhances the expression of pulmonary pro-inflammatory cytokines, whereas inhibition of AI-2 signaling attenuates the inflammatory response. Collectively, these findings suggest that dysregulation of microbiota-derived “communication signals” may contribute to amplification and imbalance of airway inflammation, particularly under conditions of infection or stress.

In addition, lipid derivatives, various fatty acids other than SCFAs, and their metabolic intermediates can also modulate pulmonary immune homeostasis. In an allergen-driven asthma model, Levan et al. (2019) reported that lipid-derived metabolites (LDMs) such as 12,13-dihydroxy-9Z-octadecenoic acid (12,13-diHOME) are elevated in the feces of high-risk individuals and may augment susceptibility to airway inflammation by regulating Treg-associated immune tolerance in the lung. Furthermore, lipid derivatives, including diacylglycerols and sphingosine-1-phosphate (S1P), are significantly dysregulated in COPD (Ding et al., 2024). Particularly in the frequent exacerbator phenotype, the levels of these lipid derivatives are closely correlated with COPD Assessment Test (CAT) scores and dyspnea severity. Collectively, these findings suggest that the aberrant accumulation of lipid derivatives may exacerbate pulmonary inflammation and immune dysregulation. Moreover, recent studies have revealed that, beyond SCFAs, patients with COPD display widespread perturbations in plasma levels of fatty acids and their intermediates, characterized by elevated levels of medium- and medium-short-chain fatty acids as well as multiple acylcarnitines (ACs), coupled with reduced concentrations of long- and very-long-chain fatty acids (LCFAs and VLCFAs) (Casadevall et al., 2025). These observations indicate that systemic energy metabolic remodeling may be an integral component of COPD pathophysiology, providing a rationale for developing metabolism-targeted therapeutic strategies.

## 4 Therapeutic potential of FMT in COPD

The systemic nature of COPD indicates that its pathophysiology extends beyond localized pulmonary inflammation and is closely linked to inter-organ signaling mediated by the gut microbiota and its metabolites. Against this backdrop, targeted modulation of gut microbial function has emerged as a promising therapeutic avenue for COPD, with FMT standing out as a key intervention. FMT, which restores microbial homeostasis by transferring gut microbiota from healthy donors, has been extensively explored in a range of chronic diseases, including inflammatory bowel disease, metabolic disorders, cancer, and neurological conditions (Guo et al., 2023; Pan et al., 2021; Chen et al., 2025; Rostgaard-Hansen et al., 2024; Ziaka and Exadaktylos, 2024). Although research on FMT in COPD is still in its initial stages, accumulating evidence supports that FMT can indirectly mitigate pulmonary inflammation and tissue injury by reshaping the gut microbial community structure and reprogramming associated metabolic profiles (Budden et al., 2024; Qiu et al., 2024). Within the framework of the gut-lung axis, the therapeutic potential of FMT likely extends beyond microbial reconstitution alone, instead involving the restoration and reprogramming of the systemic metabolite pool, with downstream effects on immune regulation, barrier integrity, and disease progression. This underscores FMT’s unique capacity to target the gut-lung axis at both microbial and metabolic levels, positioning it as a compelling candidate for COPD intervention.

### 4.1 FMT regulation of gut metabolic dysregulation

**Figure 2:**
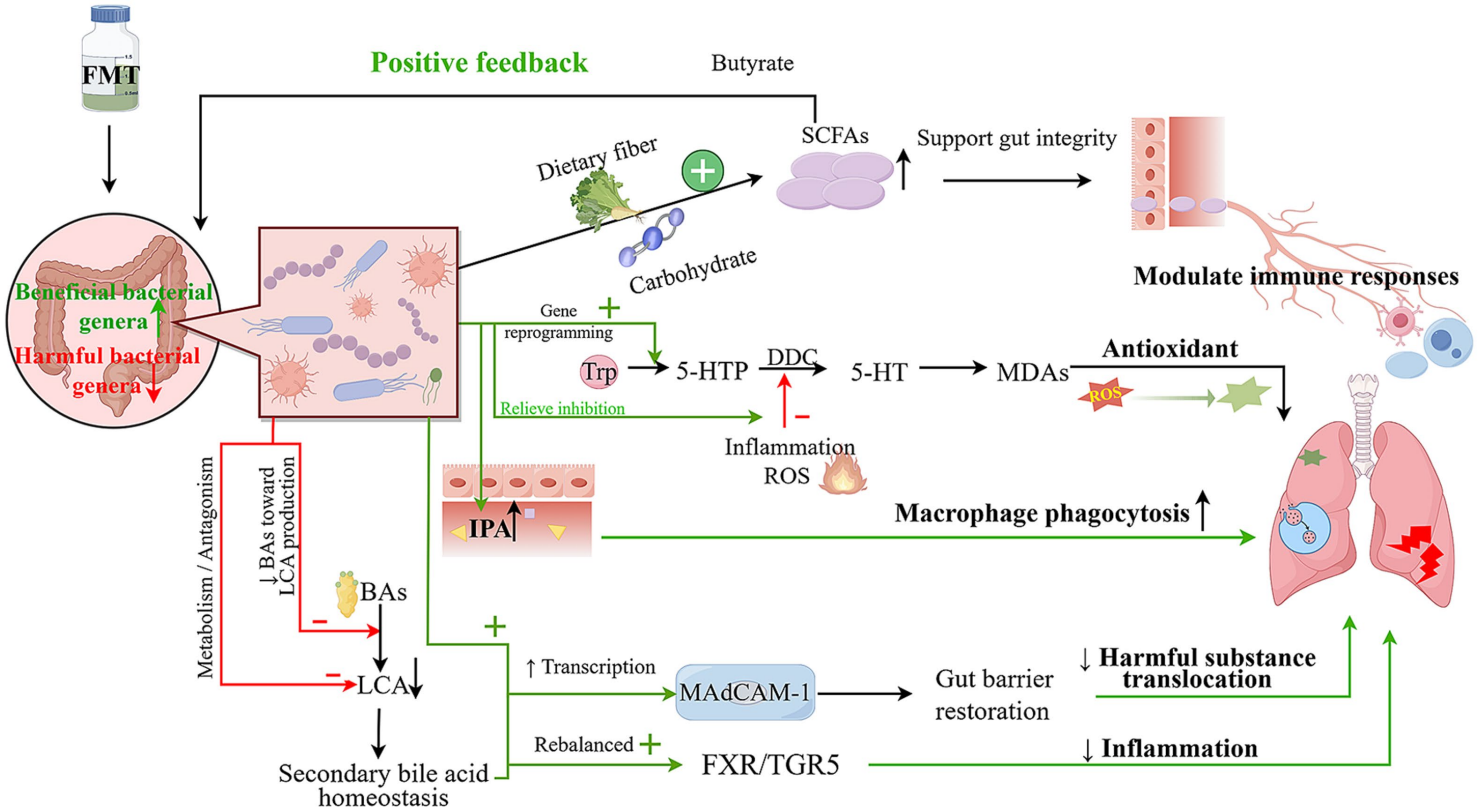
FMT modulates gut-lung axis crosstalk via microbial remodeling and metabolic re-programming to alleviate pulmonary diseases (by Figdraw). (FMT reshapes the intestinal microbial landscape by enriching beneficial bacterial genera and depleting harmful taxa, thereby initiating a cascade of metabolic reprogramming and immunomodulatory effects that converge on the gut-lung axis. Key mechanistic pathways include: short-chain fatty acid (SCFA) metabolism, bile acid (BA) homeostasis, tryptophan (Trp) metabolic reprogramming, and gut-lung axis signaling).

At the interventional level, the use of FMT combined with a high-fiber diet to attenuate the progression of emphysema represents one of the earliest attempts to directly apply FMT for improving pulmonary pathology (Jang et al., 2020). This study suggests that microbiota reconstitution, together with adequate substrate availability, may synergistically influence pulmonary outcomes and provides a translational model for FMT-based interventions in lung diseases. Subsequently, Li et al. (2021) pioneered the integration of clinical COPD sample analyses and animal experiments, confirming distinct gut microbiota dysbiosis in COPD patients, characterized by reduced diversity (especially in severe cases), increased *Firmicutes*, decreased *Bacteroidetes*, and *Prevotella* enrichment. Transplanting feces from severe COPD patients into microbiota-depleted mice induced COPD-like pathologies (pulmonary inflammation, immune dysregulation, mucus hypersecretion, airway remodeling), while healthy donor microbiota did not elicit such changes. This study provides direct causal evidence supporting the involvement of gut microbiota dysbiosis in COPD pathogenesis and establishes a theoretical foundation for the future development of FMT-based therapeutic strategies for COPD and other pulmonary diseases. Building on these findings, subsequent animal studies further validated that FMT restores gut microbial and metabolic homeostasis, partially alleviating pulmonary inflammatory and structural damage. Figure 2 schematically illustrates the integrated mechanistic network through which FMT modulates gut microbial composition, metabolic re-programming (including SCFA, bile acid, and tryptophan pathways), and gut-lung axis crosstalk to attenuate pulmonary inflammation and oxidative damage.

#### 4.1.1 FMT-SCFAs

A growing body of evidence indicates that FMT exerts pivotal regulatory effects in various inflammatory and infection-related disease models by systemically reshaping gut microbial architecture and restoring SCFA-associated metabolic capacity. Tang et al. (2024) demonstrated that FMT markedly restored intestinal levels of acetic, propionic, and butyric in mice subjected to *Klebsiella pneumoniae* infection following antibiotic-induced dysbiosis, concomitantly leading to a significant improvement in host survival. Molecular analyses revealed that FMT effectively reconstituted the disrupted gut microbial ecosystem by restoring the relative abundance of beneficial phyla, including *Firmicutes* and *Bacteroidetes*, and promoting the re-emergence of key SCFA-producing genera such as *Ruminococcus*, *Eubacterium*, and *Lachnospira*. Reconstruction of this microbial community reactivated intestinal metabolic pathways centered on dietary fiber fermentation, thereby driving endogenous production of acetate, propionate, and butyrate.

In addition, in an acute respiratory distress syndrome (ARDS) model, FMT not only significantly enriched key SCFA-producing taxa with dietary fiber fermenting capacity, such as *Muribaculaceae*, *Clostridia*_UCG-014, *Prevotella*, and *Adlercreutzia*, but also concurrently suppressed genera like *Romboutsia*, which may be involved in substrate competition or metabolic imbalance, thereby globally optimizing fermentable substrate utilization (Zhang et al., 2025). Specifically, this restoration of microbial structure triggers metabolic re-programming centered on carbohydrate fermentation, supplying essential precursors for SCFA biosynthesis. Subsequently, SCFAs, particularly butyrate, further strengthen intestinal barrier integrity, establishing a positive feedback loop that promotes the sustained colonization of SCFA-producing microbial taxa.

Beyond pulmonary disease models, FMT’s ability to restore SCFA metabolic function has been consistently corroborated in inflammatory disease models such as colitis. These studies indicate that the recovery of SCFA metabolism relies on the establishment of functionally integrated, microbiota-driven metabolic networks and exhibits distinct microbiota dependence and transferability (Zhang et al., 2025). While direct evidence for FMT-mediated SCFA restoration in COPD animal models remains limited, the convergence of metabolic improvements across diverse disease models, coupled with the persistent deficit in SCFA-related functions in COPD patients, provides a biologically plausible mechanistic rationale for FMT to modulate pulmonary inflammation and barrier repair via enhancing SCFA availability.

#### 4.1.2 FMT-AAs

Beyond SCFA metabolism, FMT-mediated modulation of AA metabolism, with a particular focus on Trp, has emerged as a burgeoning and high-priority area of investigation. Although direct clinical and preclinical studies linking FMT to AA metabolic reprogramming in COPD remain limited, the systemic regulatory effects of FMT on tryptophan-associated metabolic networks have been robustly documented in a broad spectrum of other disease models.

In a valproic acid (VPA) induced mouse model of autism spectrum disorder (ASD) (Wang et al., 2023), FMT reshaped the gut microbiota characterized by a reduction in *Bacteroides* and enrichment of *Turicibacter* and *Alistipes*, thereby driving a global rebalancing of AA-related metabolic profiles. On one hand, these microbial alterations converged on regulation of the Trp-serotonin (5-HT) pathway, manifested by transcriptional reprogramming of genes involved in colonic serotonin synthesis, transport, and receptor signaling, along with upregulation of central serotonin receptors such as Htr2c. On the other hand, untargeted serum metabolomics revealed significant alterations in AA and nitrogen metabolism-related molecules, including L-glutamate (Glu), glutathione (GSH), and oxidized L-proline, which were enriched in glutamatergic and GABAergic synaptic pathways and accompanied by upregulation of synaptic plasticity-associated genes such as *Shank1/3* and *Cacna1a*. Correlation network analyses integrated the interplay among microbial taxa, metabolites, and gene expression profiles, providing a key mechanistic framework for FMT-based COPD interventions. Specifically, FMT may rectify dysregulated AA metabolism, encompassing Trp, Glu, and GSH, via gut microbiota remodeling, thereby modulating host immune responses, oxidative stress signaling, and systemic inflammatory phenotypes, and establishing a transferable conceptual pathway for gut-lung axis metabolic interventions. Consistent evidence from other inflammatory disease models supports FMT’s regulation of Trp metabolic pathways. For instance, in a mouse model of radiation-induced enteritis, FMT alleviated intestinal epithelial injury, restored beneficial taxa (*Lactobacillaceae* and *Lachnospiraceae*), and elevated Trp pathway metabolites (Tu et al., 2024).

In the context of COPD, Liu et al. (2024) demonstrated that probiotic intervention enhances the availability of tryptophan-related metabolites and promotes their axis-specific conversion toward 5-hydroxy-L-tryptophan (5-HTP), 5-HT, and melatonin-derived antioxidants (MDAs), such as 6-OH-Mel, thereby strengthening endogenous antioxidant defenses. Mechanistically, microbial strains were shown to produce key substrates, including L-tryptophanamide and 5-HTP, with 5-HTP subsequently converted to 5-HT via aromatic L-amino acid decarboxylase (DDC). However, the activity of DDC is negatively regulated by inflammation and reactive oxygen species (ROS). Microbial supplementation partially alleviated this inhibition under inflammatory serum conditions and progressively enhanced the efficiency of 5-HT production over time, leading to improved downstream antioxidant capacity and attenuation of inflammatory injury. Theoretically, diverse, functionally complementary FMT strategies better reshape Trp metabolic networks. Similarly, in a lung injury model, Peng et al. (2025) demonstrated via FMT experiments that transplantation of fecal microbiota from manganese-carbon dots (Mn-CDs) pretreated mice markedly restored gut microbial homeostasis in septic mice, with particular enrichment of key tryptophan metabolism-associated genera, including *Clostridium* and *Bacteroides*. Concurrent serum metabolomic analyses revealed significant enrichment of the tryptophan metabolic pathway, accompanied by a pronounced increase in levels of the metabolite indole-3-propionic acid (IPA). These metabolic perturbations were tightly linked to mitigated lung injury, augmented phagocytic activity of alveolar macrophages, and anti-inflammatory polarization, highlighting the pivotal role of the microbiota-metabolite axis in mediating the therapeutic effects of Mn-CDs.

Notably, although the directionality of FMT-induced changes in *Bacteroides* abundance is not entirely consistent across disease models, convergent evidence indicates a broadly positive restorative effect on Trp metabolic pathways (Yan et al., 2024). Given that *Bacteroides* abundance is typically reduced in patients with COPD, particularly during acute exacerbations (Bowerman et al., 2020), these findings imply that restoration or targeted modulation of this genus could constitute a plausible mechanism by which microbiota-based interventions augment Trp metabolic capacity and regulate disease progression. Collectively, these lines of evidence support the notion that FMT may exert therapeutic potential in COPD and related disorders via coordinated modulation of gut microbial composition and tryptophan metabolic networks.

#### 4.1.3 FMT-BAs

The gut microbiota plays a pivotal role in preserving inflammatory homeostasis, intestinal barrier integrity, and host metabolic balance by regulating BA composition and downstream signaling pathways. To date, a growing body of evidence from diverse disease models has indirectly supported a critical role for FMT in regulating BA metabolism and mitigating inflammation. In a mouse model of nonalcoholic fatty liver disease (NAFLD), ginsenoside Re was demonstrated to systemically restructure the gut microbial landscape, enhance intestinal barrier function, and reprogram BA metabolic profiles, thereby significantly ameliorating hepatic dysfunction. Mechanistic follow-up studies confirmed that this hepatoprotective effect was highly microbiota-dependent: antibiotic-mediated depletion of intestinal microbes significantly blunted these protective benefits, whereas FMT from Re-treated donors was sufficient to recapitulate the hepatoprotective phenotype in germ-free recipient mice (Zheng et al., 2025). Similar microbiota-dependent mechanisms have been corroborated in inflammatory bowel disease (IBD) models (Zang et al., 2025). In a dextran sulfate sodium (DSS)-induced mouse model of ulcerative colitis (UC), FMT from oleuropein (OLE)-treated donors markedly restructured the intestinal microbiota and elevated levels of anti-inflammatory BAs such as hyodeoxycholic acid (HDCA) and UDCA, thereby attenuating DSS-induced colitis.

Fidelle et al. (2023) further dissected the intrinsic mechanisms underlying BA metabolic homeostasis restoration by FMT. Their work revealed that FMT suppresses or displaces *Enterocloster* species, which tend to expand aberrantly post-antibiotic exposure, thereby mitigating the skewed impact of these taxa on BA biotransformation and curbing the generation of inhibitory secondary bile acids like lithocholic acid (LCA). Concurrently, FMT may introduce microbial taxa with the capacity to metabolize or antagonize LCA, which synergistically facilitates a further decline in LCA levels. Collectively, these alterations reorient the BA pool from a profile dominated by inhibitory secondary bile acids toward one that approximates physiological homeostasis, while concomitantly upregulating the transcription of mucosal addressin cell adhesion molecule-1 (MAdCAM-1) in endothelial cells. Furthermore, immunostimulatory taxa engrafted via FMT, such as *Akkermansia muciniphila* strain p2261, also augment MAdCAM-1 transcription. As a ligand for α4β7 integrin, elevated MAdCAM-1 expression promotes the retention of α4β7⁺ Treg17 cells within the intestinal lumen, thus limiting their ectopic migration into the tumor microenvironment.

This mechanistic paradigm offers critical insights into the potential utility of FMT for COPD management. Prior investigations have established that COPD is commonly associated with compromised intestinal barrier integrity, thereby facilitating the translocation of bacterial components and inflammatory mediators (Wu et al., 2025; Lv et al., 2025). In this context, the reconstitution of targeted microbial consortia to restore gut microbial homeostasis and strengthen the intestinal immune barrier may sequester proinflammatory immune subsets (e.g., Th17 cells) within the intestinal compartment, preventing their unregulated trafficking to the lung and subsequent contribution to chronic inflammatory cascades. Concurrently, the restoration of secondary BA homeostasis may re-equilibrate FXR-TGR5 signaling cascades, thereby attenuating inflammatory signal amplification and mitigating pulmonary inflammatory damage (Guo et al., 2016; Gadaleta et al., 2011). Furthermore, these findings suggest that circulating soluble MAdCAM-1 concentrations may represent a promising biomarker for gauging intestinal barrier integrity and predicting therapeutic efficacy, opening new avenues for personalized immunomodulatory strategies in COPD.

Given the shared mucosal immune system and the established gut-lung axis, it is plausible that similar BA-mediated anti-inflammatory mechanisms could operate in the pulmonary environment of COPD patients. Nevertheless, substantial evidence gaps remain in the current understanding of BA metabolism in the context of COPD. On the one hand, disease-specific studies directly investigating the causal relationship between BA dysregulation and COPD initiation or progression are scarce. Most mechanistic evidence linking bile acid signaling to immune and inflammatory regulation derives from models of inflammatory bowel disease or liver disorders, and its applicability within the COPD-specific pathological milieu remains to be established. On the other hand, studies directly evaluating the impact of FMT on BA metabolic profiles and associated immunological effects in patients with COPD are currently lacking. Although the central role of BAs in immune regulation, the regulatory framework of the gut-lung axis, and indirect evidence from other disease models collectively allow the preliminary inference that FMT may modulate gut-lung axis inflammation and maintain pulmonary immune homeostasis through restoration of the BA-FXR/TGR5 signaling axis, this concept currently remains theoretical and lacks COPD-specific empirical validation. Therefore, future studies are urgently needed to systematically characterize BA metabolic disturbances in patients with COPD and elucidate their pathological significance, as well as to define the precise effects of FMT on bile acid pool composition and pulmonary immune regulation, thereby providing direct and robust experimental and clinical evidence to substantiate these hypotheses. Under this foundation, integration of multi-omics bile acid profiling, functional microbiota annotation, and pulmonary immune phenotyping within a unified research framework may facilitate the transition of FMT from a mechanistic hypothesis toward a testable, stratifiable, and precision-based therapeutic strategy.

#### 4.1.4 Systemic and multi-pathway effects of FMT

**Figure 3:**
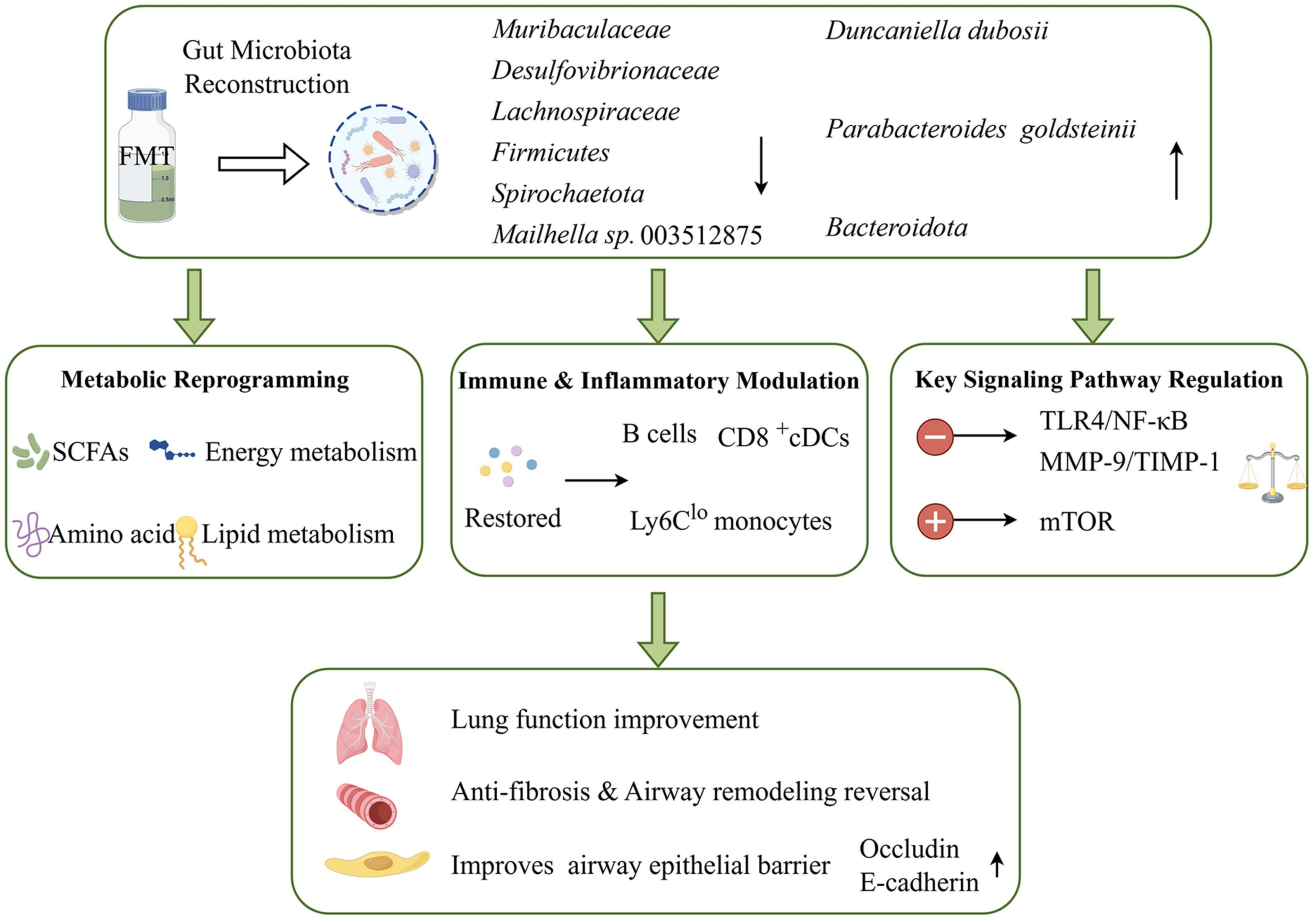
FMT reconstructs gut microbiota to modulate metabolic, immune, and signaling pathways, ameliorating pulmonary pathophysiology in COPD. (By Figdraw.) (This schematic details the integrated, multi-modal mechanisms through which FMT alleviates COPD-associated pulmonary dysfunction via gut microbiota reconstruction. Key mechanistic cascades include: metabolic reprogramming, immune & inflammatory modulation, and key signaling pathway regulation).

Collective evidence from studies of SCFAs, AAs, and BAs demonstrates that gut metabolic perturbations in COPD are not restricted to a single pathway but instead represent concurrent dysregulation across multiple interconnected metabolic axes. These pathways are intricately linked in regulating immune responses, preserving barrier homeostasis, and amplifying inflammatory signaling, together forming reinforcing positive feedback loops that perpetuate disease pathology. Accordingly, the therapeutic effects of FMT are unlikely to stem from restoring a single metabolite class, but rather from reorganizing the global gut metabolic network to elicit cross-pathway, systems-level biological effects. Figure 3 schematically illustrates this multi-modal mechanism, showing how FMT reconstructs gut microbial communities to drive metabolic reprogramming, immune modulation, and signaling pathway regulation, ultimately improving lung function in COPD.

In an FMT-treated rat model of ARDS, Zhang et al. (2025) demonstrated that FMT not only restored canonical SCFAs, such as butyrate and propionate, but also concomitantly modulated multiple AA metabolic pathways, including arginine-proline metabolism, as well as lipid metabolic pathways encompassing glycerophospholipid and sphingolipid metabolism. These findings directly indicate that FMT exerts a pronounced capacity for metabolic reprogramming rather than merely correcting a single class of metabolic perturbations. Further transcriptomic analyses revealed that FMT additionally modulates the mTOR signaling pathway, cytokine-cytokine receptor interaction pathways, and multiple immune response-related signaling networks, thereby enhancing anti-inflammatory responses and promoting alveolar epithelial repair. Consistently, Budden et al. (2024) reported that FMT confers multilayered protective effects in experimental models of COPD. At the microbial level, FMT markedly reversed cigarette smoke-induced gut dysbiosis, suppressing the pathological enrichment of taxa such as *Muribaculaceae*, *Desulfovibrionaceae*, and certain members of *Lachnospiraceae*, while promoting colonization by beneficial anaerobes including *Duncaniella dubosii* and *Parabacteroides goldsteinii*. This was not only strongly associated with disease phenotypes but also constrained the expansion of opportunistic pathogens, such as *Mailhella* *sp.*003512875, through competitive exclusion mechanisms, reflecting a multitargeted and network-based mode of microbiota regulation. At the functional level, multi-omics analyses indicated that cigarette smoke exposure impaired microbial metabolic capacity, characterized by downregulation of glucose and starch metabolism pathways, reduced AAs-derived metabolites, and aberrant accumulation of secondary bile acids. FMT partially restored these metabolic pathways and upregulated key proteins involved in antioxidative stress responses and energy metabolism, thereby reshaping the microbial metabolite landscape to provide anti-inflammatory and antioxidative support to the host and reinforce intestinal barrier integrity and immune homeostasis. At the immunological level, FMT concurrently alleviated pulmonary inflammation and alveolar structural damage, mitigated colonic immune injury, and suppressed Toll-like receptor-associated signaling, while systemically reshaping the peripheral immune landscape to restore the homeostasis of key immune subsets, including B cells, CD8⁺ conventional dendritic cells, and non-classical monocytes. Collectively, these lines of evidence indicate that FMT does not act via a single molecular target. Rather, it orchestrates microbiota-metabolism-immunity axes to achieve multilayered and systemic amelioration of lung injury.

Beyond global metabolic network reprogramming, Xu et al. (2025) further elucidated the multitiered regulatory effects of FMT on key structural injury processes in COPD at the level of specific pathological pathways. Focusing on the MMP-9/TIMP-1 axis, which is a pivotal molecular pathway governing airway remodeling and fibrosis, the study demonstrated that in a cigarette smoke plus LPS-induced rat model of COPD, the expression of MMP-9 in lung was markedly upregulated and the regulation of TIMP-1 was imbalanced, resulting in the coexistence of excessive extracellular matrix degradation and aberrant deposition, thereby driving airway wall thickening and fibrotic progression. Following FMT intervention, the recovery of gut microbial diversity, manifested by elevated *Bacteroidetes* abundance and relative declines in *Firmicutes* and *Spirochaetota* taxa, was accompanied by potent inhibition of aberrant TLR4/NF-κB signaling cascades, as well as the downregulation of dysregulated MMP-9 and TIMP-1 expression. Concurrently, FMT enhanced the expression of epithelial junctional proteins (e.g., E-cadherin and occludin), thus reinforcing airway epithelial barrier integrity. Collectively, these alterations alleviated pulmonary inflammation, reduced collagen deposition, and yielded notable improvements in key lung function metrics (inspiratory capacity [IC], forced vital capacity [FVC], dynamic compliance [Cdyn]). These findings underscore that FMT can directly modulate airway remodeling and fibrosis by targeting the microbiota-immunity-protease balance axis.

Collectively, the mechanistic actions of FMT in respiratory diseases display marked multi-pathway synergistic properties. On one hand, FMT restructures the gut microbial landscape, restoring metabolic homeostasis across diverse classes of microbiota-derived metabolites (SCFAs, AAs, BAs). On the other hand, FMT modulates pivotal signaling pathways (mTOR, TLR4/NF-κB, MMP-9/TIMP-1 axis), exerting systemic effects on immune regulation, barrier repair, inflammation suppression, and tissue structural remodeling. These findings indicate that future investigations and clinical applications of FMT should transcend a reductionist focus on individual metabolites or isolated signaling pathways. Instead, a systems-level framework emphasizing coordinated reconstruction of metabolic networks and the immune-tissue structural axis may yield more stable and durable therapeutic strategies for complex respiratory diseases like COPD.

### 4.2 Safety and translational challenges of FMT intervention

At present, direct clinical evidence supporting the use of FMT for the treatment of COPD remains limited. Available supportive data are largely indirect, deriving from COPD-related preclinical animal studies, clinical experience with FMT in other diseases, and microbiota-based interventions such as probiotics and prebiotics in COPD. Previous studies suggest that selected probiotics, including *Lactobacillus* and *Bifidobacterium* species, when combined with standard therapy, may improve lung function and reduce inflammatory markers in patients with COPD, accompanied by measurable alterations in gut microbiota composition (Nicola et al., 2024; Chen et al., 2024; Liu et al., 2024). These findings indirectly support the feasibility of modulating the gut microbiome to influence COPD-related outcomes. However, such evidence cannot be directly extrapolated to the therapeutic effects of FMT. A cross-sectional analysis by Hong and Luo (2024) based on data from the U. S. National Health and Nutrition Examination Survey (NHANES 2007–2012) reported a significant association between the intake of probiotics, prebiotics, or yogurt and a lower prevalence of COPD (OR = 0.75, 95% CI 0.57–0.98). This observation provides population-level evidence in support of the gut-lung axis and suggests a potential protective role of gut microbiota-targeted interventions in COPD. Nevertheless, this observational design precludes causal inference. Moreover, the independent effects of probiotics, prebiotics, and yogurt were not distinguished, and more direct microbiota interventions such as FMT were not assessed. Consequently, this study offers only preliminary clues regarding the association between gut microbiota and COPD risk. Its conclusions require validation through prospective cohort studies or randomized controlled trials, with particular emphasis on the clinical efficacy and mechanisms of FMT in COPD. Although an increasing number of basic and preclinical studies indicate that the gut microbiota and its derived metabolites play important regulatory roles in systemic inflammation and immune dysregulation in COPD, no published original clinical studies have yet directly applied FMT in COPD patients while systematically evaluating its efficacy and safety.

This evidence gap does not negate the potential value of FMT in COPD, but rather reflects the multiple challenges associated with its clinical translation in this disease context. First, COPD is characterized by pronounced clinical heterogeneity and a dynamic disease course. Substantial inter-individual variability exists in inflammatory phenotypes, metabolic status, exacerbation frequency, and comorbidity profiles, making it difficult for a uniform FMT intervention to achieve consistent efficacy in unstratified patient populations. In addition, the long-term or repeated use of antibiotics, corticosteroids, and bronchodilators in COPD patients profoundly alters gut microbiota structure and function, potentially attenuating or masking the true effects of FMT and further increasing uncertainty in efficacy assessment. Second, safety considerations are particularly critical in the COPD population. Most patients with COPD are older adults and frequently present with frailty, malnutrition, and multiple systemic comorbidities, and with a markedly increased risk of infection during acute exacerbations. In this context, FMT, as an intervention involving the transfer of live microbial communities, requires rigorous evaluation of potential pathogen transmission, immune-related adverse events, and long-term safety under strict inclusion criteria and enhanced monitoring frameworks, yet safety data specific to COPD remain scarce. Third, the efficacy of FMT is highly dependent on donor characteristics and preparation protocols. However, optimal donor selection criteria and reproducible functional indicators specific to COPD, such as enrichment of SCFA-producing taxa or microbiota with defined metabolic advantages, have yet to be established (Ianiro et al., 2022; Paramsothy et al., 2019). Establishing such standards will require large-scale COPD-specific datasets linking microbial composition, metabolic profiles, and clinical outcomes. Moreover, donor-recipient microbiota compatibility, dietary and pharmacological exposures, and host genetic background may collectively determine post-transplant functional reconstitution. Identifying patients most likely to benefit and developing predictive models are prerequisites for precision microbiota-based interventions, yet current evidence remains largely theoretical. Fourth, routes of administration and regulatory-ethical considerations represent additional challenges. Conventional FMT delivery methods, such as colonoscopic infusion or nasogastric administration, are invasive and may limit acceptability in chronic respiratory diseases. In recent years, non-invasive strategies such as oral FMT capsules have demonstrated improved feasibility in other indications, suggesting potential for further clinical translation (Yu et al., 2025). Nevertheless, as a live biotherapeutic product, FMT requires continued refinement of quality control, regulatory pathways, and ethical frameworks, and optimized administration strategies for the COPD population remain insufficiently studied.

Notably, although published clinical evidence remains limited, the registration of multiple FMT studies targeting COPD across global platforms marks a pivotal shift from theoretical feasibility to clinical evaluation (Table 2). These ongoing trials vary in focus, ranging from addressing nutrient uptake and metabolic homeostasis in malnourished COPD patients to evaluating the restoration of gut-lung microecological balance in acute exacerbation of chronic obstructive pulmonary disease (AECOPD), and utilizing standardized washed microbiota transplantation (WMT) for patients with COPD and pneumonia. However, the most critical evidence gap remains the need for large-scale, multicenter, randomized controlled trials in COPD populations. Such studies should systematically assess the impact of FMT on key clinical endpoints, including lung function, exacerbation rate, and quality of life, while concurrently evaluating gut microbiota, metabolomic, and immunologic readouts to determine whether effects are mediated via the gut-lung axis and key metabolic pathways. Future studies should incorporate patient stratification based on inflammatory phenotype, metabolic profile, or baseline microbiome features. Furthermore, comparing the safety and feasibility of different administration routes (e.g., oral capsules, nasojejunal, nasointestinal, or colonic catheterization) under strict donor screening is essential to lay the groundwork for integrating FMT into comprehensive COPD management.

**Table 2:** Registered clinical trials of FMT in COPD.

Number	Registry platform	Disease	Category	Route of administration	Primary endpoint	Potential mechanistic	Notes
NCT04861649	ClinicalTrials.gov	COPD with malnutrition	FMT	Nasointestinal tube	nutrient uptake improvement	Modulation of the metabolite-neuroendocrine axis to restore insulin and glucolipid metabolic homeostasis	Focus on gut-microbiota-mediated nutrient assimilation
ChiCTR2500110513	ChiCTR	AECOPD		Nasojejunal or colonic catheterization	Efficacy and safety	Restoration of gut-lung microecological homeostasis and modulation of immune-inflammatory responses via the gut-lung axis	Pre-registration; AECOPD setting; safety critical
ChiCTR2400092352		COPD and pneumonia	WMT	—	Improvement in lung function	Modulation of the gut-lung axis to influence pulmonary function and ventilation capacity	Retrospective registration; WMT standardized; suitable for clinical translation

## 5 Conclusion and perspectives

Current evidence indicates that COPD is not merely a localized pulmonary inflammatory disease, but a multidimensional syndrome characterized by systemic inflammation, metabolic remodeling, and multi-organ dysfunction. Within the gut-lung axis framework, dysbiosis of the gut microbiota and concomitant metabolic dysfunction have emerged as pivotal transducers linking intestinal ecological perturbations to chronic pulmonary inflammation. Studies integrating SCFAs, AAs (notably Trp), and BAs reveal that COPD patients commonly exhibit functional microbiota disturbances, primarily characterized by the depletion or dysregulation of the bioactive metabolite pool. These metabolic perturbations are not isolated; instead, they converge upon immune regulation, barrier homeostasis, and oxidative stress defense pathways, collectively amplifying chronic pulmonary inflammation and tissue injury.

FMT, as an intervention capable of comprehensively remodeling gut microbial structure and function, offers potential benefits that transcend the mere correction of compositional abnormalities. Its therapeutic potency is likely predicated on the systemic restoration of disrupted metabolic pathways and metabolite supply. Evidence from animal studies and trans-disease models suggests that FMT can reconstitute SCFA-producing microbiota and recalibrate Trp and BA metabolic networks. This, in turn, restores immune balance, fortifies barrier function, and mitigates oxidative stress, collectively modulating pulmonary inflammatory outcomes via the gut-lung axis. However, it is imperative to note that FMT studies in COPD remain largely limited to preclinical and indirect evidence. Its efficacy, safety, and target population have yet to be systematically validated in high-quality clinical trials.

Based on the current state of research, several critical challenges remain to be addressed to bridge the gap between bench and bedside. First, regarding mechanisms, research must pivot from descriptive differences in microbial composition toward a function-oriented framework. Particular attention should be devoted to the dynamic flux of specific metabolic pathways and key metabolites across different stages and phenotypes of COPD, as well as their causal roles in immune regulation and oxidative stress. Second, regarding methodology, the integrative application of multi-omics approaches, including metagenomics, metabolomics, transcriptomics, and immune phenotyping, will facilitate the construction of a holistic microbiota-metabolite-host response network, thereby bolstering the validity of mechanistic inference. Third, regarding intervention strategy, future studies should further disentangle the relative contributions of microbial structural reconstruction and metabolite restoration. This distinction is crucial to ascertain whether more precise and controllable strategies, such as defined functional consortia, targeted metabolite supplementation, or next-generation probiotics, can replicate comparable therapeutic benefits. Moreover, given the marked clinical heterogeneity of COPD, disease phenotypes, comorbidity profiles, and lifestyle factors (e.g., smoking, diet) may profoundly modulate gut microbial composition and metabolic function. Accordingly, future studies should emphasize patient stratification and individual variability to identify subpopulations most likely to benefit from microbiota- or metabolism-targeted interventions. Finally, the long-term safety, reproducibility, and procedural standardization of FMT and related microbiota-based interventions must be validated through well-designed prospective randomized controlled trials before widespread clinical translation.

Taken together, gut microbiota-derived metabolites represent a central regulatory axis linking the gut and lung, providing a novel conceptual framework for understanding the systemic pathophysiology of COPD. As a strategy capable of globally reconfiguring microbial metabolic function, FMT shows potential research value in COPD intervention. However, its clinical translation requires a prudent approach, grounded in elucidated mechanisms, robust evidence, and precision-oriented strategies. Future studies focusing on the restoration of the metabolite pool as a quantifiable therapeutic target may unlock new avenues for the integrated management of COPD.
